# Supplementary material for: Epigenetic Treatment Alters Immune-Related Gene Signatures to Increase the Sensitivity of Anti PD-L1 Drugs
Source: Cancers (Basel). 2025 Jul 23;17(15):2431. doi: 10.3390/cancers17152431 (PMC12345851; doi:10.3390/cancers17152431)
Supplement: Supplementary file 1 [file cancers-17-02431-s001.zip › supple figure 1 and 2A2B.pdf]

Figure S1

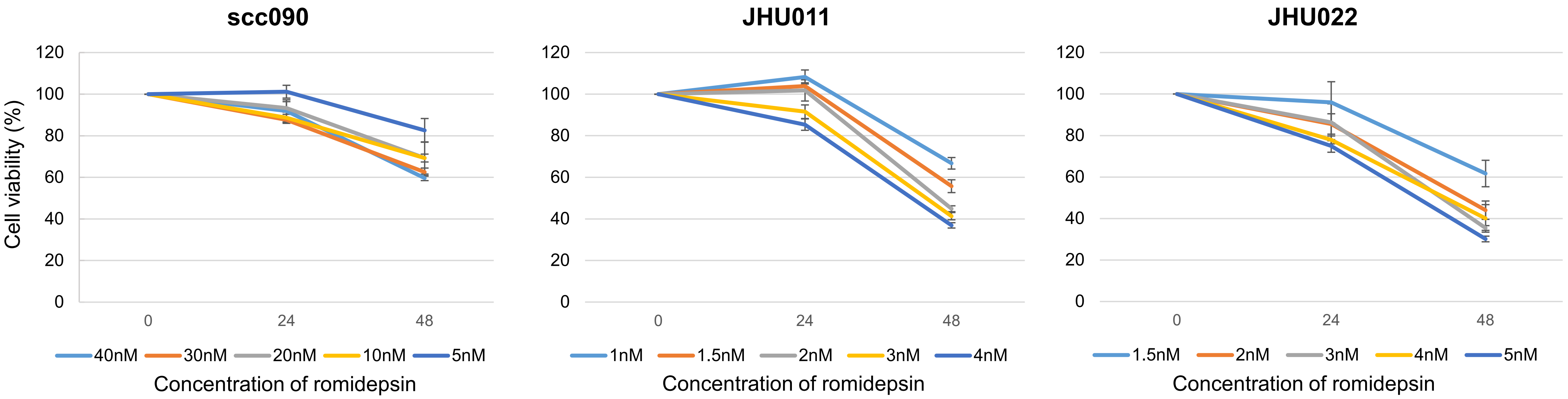
scc090
JHU011
JHU022
Cell viability (%)
120
100
80
60
40
20
0
0
24
48
40nM
30nM
20nM
10nM
5nM
1nM
1.5nM
2nM
3nM
4nM
1.5nM
2nM
3nM
4nM
5nM
Concentration of romidepsin

Figure S2A

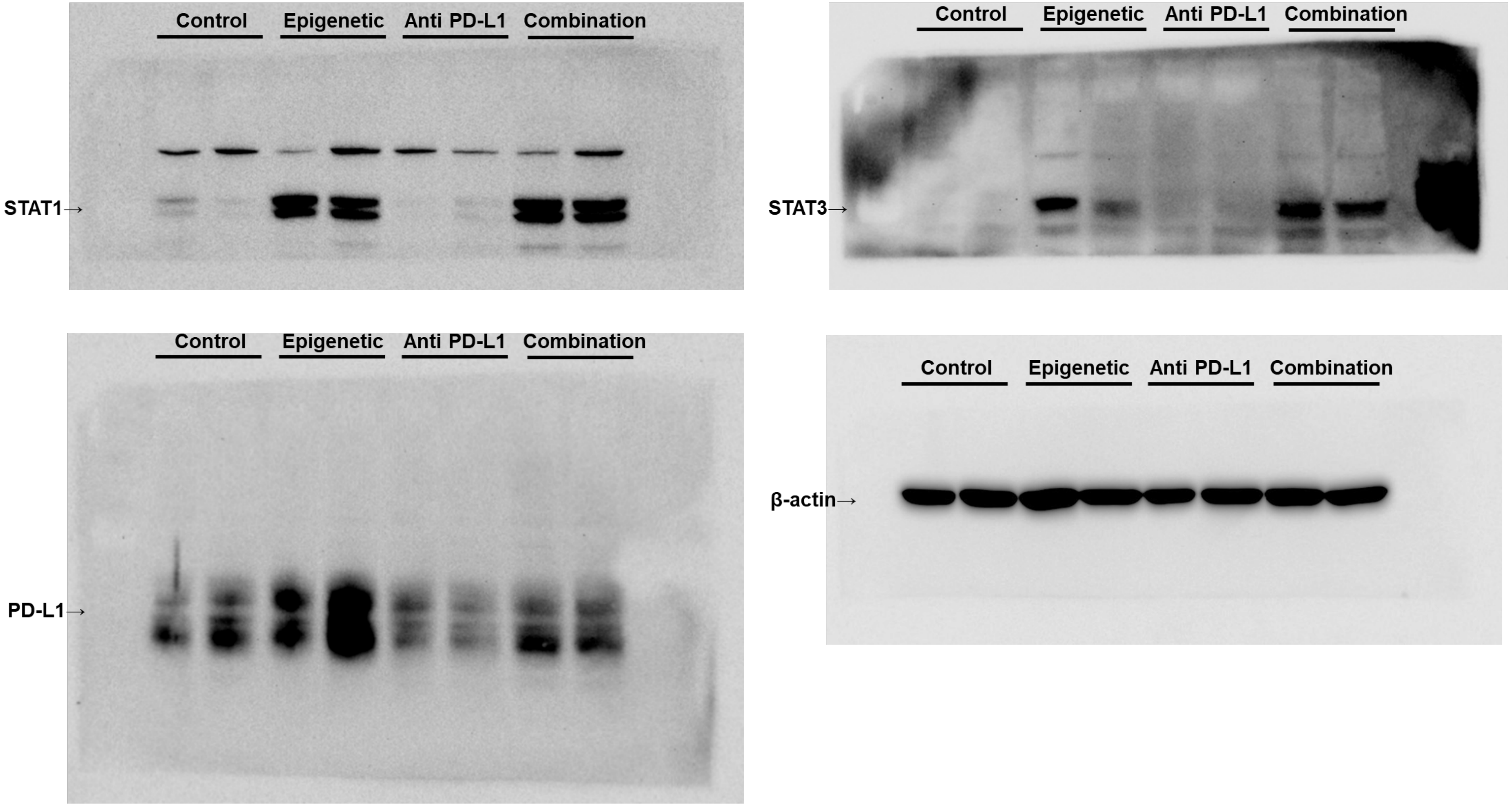
Control
Epigenetic
Anti PD-L1
Combination
STAT1→
Control
Epigenetic
Anti PD-L1
Combination
STAT3→
Control
Epigenetic
Anti PD-L1
Combination
PD-L1→
Control
Epigenetic
Anti PD-L1
Combination
β-actin→

Figure S2B

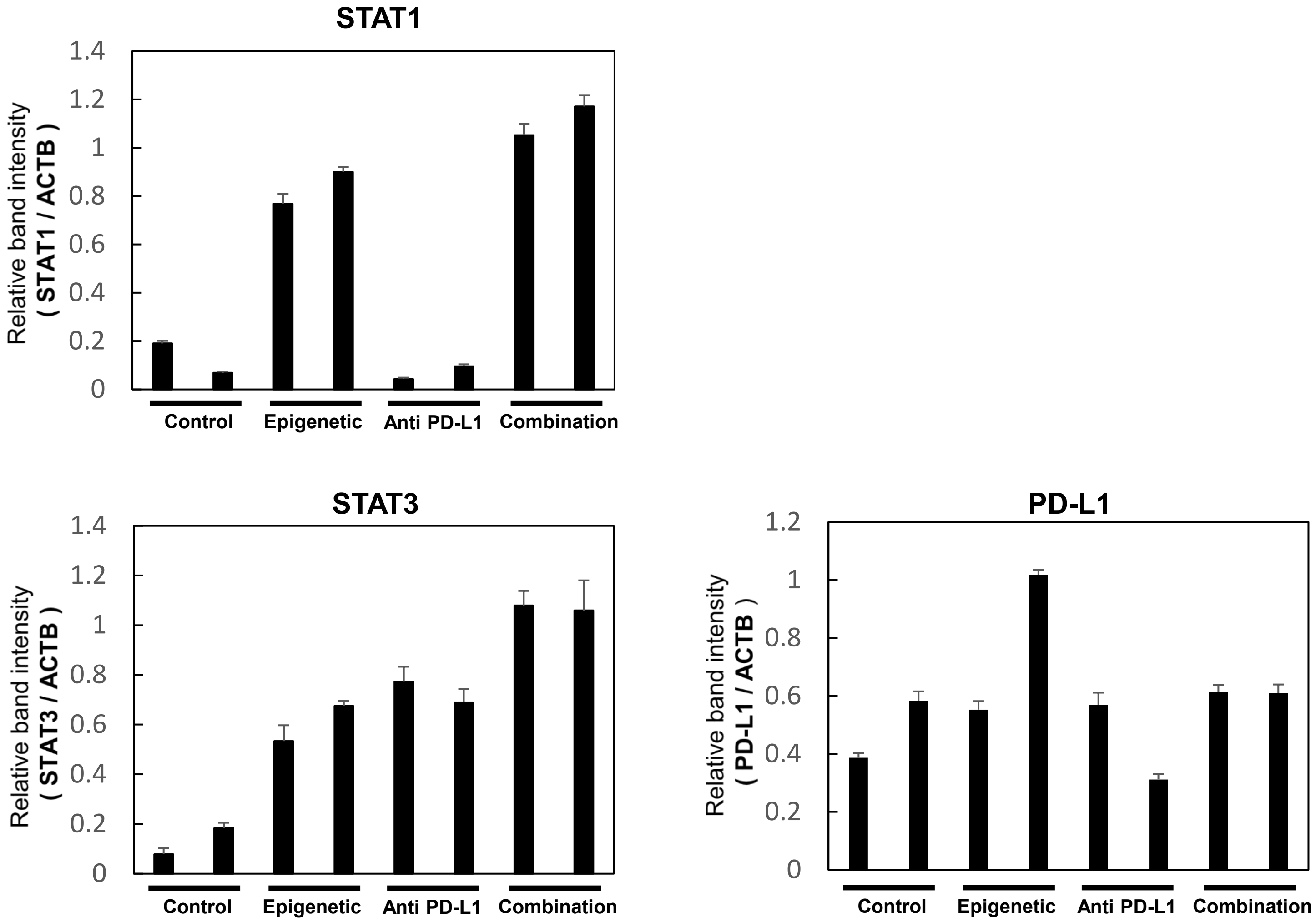

The bars are the mean ± S.D. of relative band intensity per ACTB. Densitometry readings of each protein are measured in three times.
